# Prevalence of vulvovaginitis and bacterial vaginosis in patients with koilocytosis

**DOI:** 10.1590/S1516-31802008000600008

**Published:** 2008-11-06

**Authors:** Ana Claudia Camargo Campos, Ruffo Freitas-Junior, Luiz Fernando Jubé Ribeiro, Régis Resende Paulinelli, Cleomenes Reis

**Keywords:** Vulvovaginitis, Vaginosis, bacterial, Candidiasis, Trichomonas infections, Papillomaviridae, Vulvovaginite, Vaginose bacteriana, Candidíase, Tricomoníase, Papillomaviridae

## Abstract

**CONTEXT AND OBJECTIVE:**

Empirical discussion regarding an association between koilocytosis and vulvovaginitis often occurs. Thus, the objective of this study was to assess the prevalence of microorganisms associated with bacterial vaginosis and vulvovaginitis in women with and without koilocytosis.

**DESIGN AND SETTING:**

Analytical cross-sectional study including two cohorts of women (with and without koilocytosis) who attended a cancer hospital in the city of Goiânia, state of Goiás.

**METHODS:**

A total of 102 patients entered the study. The whiff test, Gram and Papanicolaou staining and bacterial and fungal culturing were performed. The results were observed using univariate analysis. The odds ratio and confidence interval (CI) of the variables were calculated; P-values < 0.05 were considered significant.

**RESULTS:**

The prevalence of bacterial colonization was similar in patients with and without koilocytosis. The odds ratio for candidiasis was 1.43 (CI 1.05-1.95) and the odds ratio for trichomoniasis was 1.78 (CI 1.49-2.12), in patients with koilocytosis.

**CONCLUSIONS:**

The prevalence of candidiasis and trichomoniasis seems to be higher in patients with koilocytosis.

## RESUMO

**CONTEXTO E OBJETIVO:**

Existe uma discussão empírica sobre a associação de coilocitose e vulvovaginite. Assim, o objetivo deste estudo foi saber a prevalência de microorganismos associados à vaginose bacteriana e à vulvovaginite em mulheres com e sem coilocitose.

**DESENHO E LOCAL:**

Estudo transversal analítico, que incluiu duas coortes de mulheres, com e sem coilocitose, atendidas em um hospital de referência de câncer, na cidade de Goiânia, estado de Goiás.

**MÉTODOS:**

Um total de 102 pacientes entrou no estudo. Foram feitos os testes whiff, Gram e Papanicolaou, além de cultura para bactérias e fungos. Utilizou-se análise univariada, com o cálculo do risco relativo e do intervalo de confiança (IC). Considerou-se significativo quando valor de P < 0,05.

**RESULTADOS:**

A prevalência de colonização bacteriana foi similar em pacientes com e sem coilocitose. O risco relativo para candidíase observado em mulheres com coilocitose foi de 1,43 (IC 1,05-1,95) e, para tricomoníase, 1,78 (IC 1,49-2,12).

**CONCLUSÃO:**

A prevalência de candidíase e de tricomoníase parece ser maior nas pacientes com coilocitose.

## INTRODUCTION

Infectious processes in the vagina caused by potentially pathogenic microorganisms are very common and are the result of growing profusion of infectious or saprophytic agents.^1^ Genital infections are generally caused by protozoa, yeast, bacteria and viruses that cause increased vaginal secretion, irritation and vulvar pruritus, and sometimes bad smells. Evaluation of the vaginal secretions under the microscope may assist in arriving at the correct diagnosis, but it is better to obtain confirmation by culturing, since non-infectious inflammatory processes may present similar symptomatology.^1,2^

The autochthonous vaginal flora is complex and its composition varies according to a multiplicity of events such as hormonal factors; multiple partners; use of oral contraceptives; use of antibiotic therapy for infections occurring in other locations; trichomoniasis, candidiasis and other sexually transmitted diseases; diabetes; use of condoms; smoking; and bad habits regarding hygiene. All these are considered to be risk factors for the appearance of genital infections.^3,4^

Lesions induced by the human papillomavirus (HPV) are usually associated with vaginal infections. Among the various microbiological studies regarding genital infections, few in the literature have related the presence of koilocytosis to infections by other microorganisms.^5^

## OBJECTIVE

The objective of the present study was therefore to determine the prevalence of vulvovaginitis caused by *Trichomonas vaginalis, Candida* sp. and bacterial vaginosis in women with and without koilocytosis.

## METHODS

After approval by the Ethics Committee for Human and Animal Research of Hospital das Clínicas (HC), Universidade Federal de Goiás (UFG), in Goiânia, 102 female patients aged 21 to 58 years from a Breast and Gynecology Service were included in the study. The sample size was calculated to detect a prevalence of koilocytosis of 35% or higher, with a confidence interval of 95% and an error of 10% (0.10). A total of 88 patients would be required, but we adjusted this upwards by 15% in order to allow for losses of patients. Thus, a total of 102 patients was achieved.

For all the women, the results from their last Pap test carried out prior to the present study were verified. This was the information used to divide the patients into groups with and without koilocytosis. Thus, 60 patients with koilocytosis were enrolled. The negative group included 42 patients within the same age group, without cytological signs of lesions. After the patients had received information about the study and had consented to their participation, material was collected from them and sent to the Microbiology Laboratory of the Instituto de Patologia Tropical e Saúde Pública (IPTSP), UFG.

The women were allocated to the positive group if their diagnosis had been confirmed by cytology, clinical colposcopy and biopsy.^6^ None of the patients evaluated had been using topical or systemic antibiotics during the six months preceding the study.

Vulvovaginitis with a solely clinical diagnosis was characterized by a complaint of at least one of the following symptoms: vulvar pruritus, dyspareunia, external dysuria or vaginal burning, and the presence of clinical signs suggestive of vulvovaginitis, such as vulvar hyperemia, vulvar fissures, vaginal hyperemia or leukorrhea.^7^ For bacterial vaginosis to be defined, three of the four clinical criteria established needed to be present: leukorrhea, pH greater than 4.5, positive amine test and presence of "clue cells".^3^ The vaginal pH was measured while performing the examination with the speculum. Vaginal secretions were collected from the base of the posterior vaginal fornix, the endocervix and the ectocervix, and smears were put onto glass slides for examination under the microscope. The whiff test^8^ and staining for the Gram and Papanicolaou tests were done on these smears.

The vaginal secretion smears stained using the Gram method were examined and evaluated in accordance with the classification of Nugent et al.^9^ With the aid of a swab, a sample was collected from the base of the posterior vaginal fornix and put into a tube containing brain heart infusion (BHI) broth and BHI prereduced anaerobically sterilized (PRAS) broth. The latter is a prereduced medium containing hemin, vitamin K and yeast extract for isolation of anaerobes and microaerophiles that is sterilized under anaerobiosis and incubated in a glass chamber for 24 hours at 36.5 °C for enrichment.^8^

The bacteria were isolated by means of MacConkey agar, mannitol and sowing on three agar gel bases: one enriched with 5% sheep blood and incubated at 36.5 °C for 24 to 48 hours; another enriched with 5% human blood and incubated at 36.5 °C for 24 to 48 hours; and the third enriched with 5% sheep blood supplemented with hemin and vitamin K and incubated at 36.5 °C for 72 hours.^4^ After isolation, the bacteria were identified by means of biochemical and enzymatic tests for colonies of enterobacteria, performed in MacConkey medium: triple sugar iron, glucose, lactose, sucrose, mannitol, indole, motility, citrate, urea, phenylalanine, decarboxylase (lysine, ornithine and arginine), methyl red and Voges-Proskauer.^4^

To identify Gram-positive bacteria, the following tests were utilized: catalase, superficial and deep hemolysis on agar with 5% sheep blood, mannitol, coagulase, DNase, bacitracin, novobiocin, optochin, nitrate reduction, Voges-Proskauer, glucose, threalose, citrate, mannose, xylose, sucrose, fructose, oxidase and ornithine decarboxylase.^4^

To isolate the strictly anaerobic bacteria and microaerophiles, an anaerobiosis jar was used. Identification was accomplished by means of the following biochemical tests: ramnose, threalose, xylose, sucrose, mannitol, maltose, glucose, lactose, gelatinase, esculin, urea, methyl red, motility and indole.^4^

The yeasts in the vaginal secretion samples were isolated on Sabouraud agar, incubated at room temperature for 15 days.^10,11^ Characteristic colonies were subjected to identification tests consisting of formation of germinative tubes in the presence of fetal bovine serum. These appeared as fine cylindrical filaments, with production of chlamydoconidia on cornmeal agar. Biochemical tests for carbohydrate assimilation were performed, such as glucose, raffinose, cellobiose and threalose, observing the growth around this source offered as a solid medium, following incubation at 30 °C for 24 hours.^10^

*Trichomonas vaginalis* was diagnosed when a unicellular microorganism of ovoid or rounded shape with pale or grayish cytoplasm was observed. It could also present granules at its center and a vesicular shape. Diagnoses were made under the microscope, on fresh samples.^12^

For the statistical analysis, the chi-squared (χ^2^), Fisher exact and Student t tests were used. Odds ratios (ORs) were expressed with a 95% confidence interval, and p values ≤ 5% were considered to be significant.^13^ The software used for the statistical analysis was the Statistical Package for the Social Sciences (SPSS), version 11.0.1. Subanalyses of the study group were not planned, because of the small sample size.

## RESULTS

The patients’ mean age was 34.9 years (± 8.86) in the group with koilocytosis and 38.9 years (± 12.64) in the group without such lesions (P = 0.08).

Among the 102 patients assessed, bacteria were isolated from 92 of them and 136 microorganisms were identified. Ten patients presented clinical and laboratory signs indicative of bacterial vulvovaginitis. The species of staphylococci isolated and identified in the two groups were the following: *Staphylococcus aureus* 22.79%; *Staphylococcus haemolyticus* 8.08%; *Staphylococcus xylosus* 5.88%; *Streptococcus agalactiae* 5.14%; *Staphylococcus sciuri* 2.94%; *Staphylococcus epidermidis, Staphylococcus saprophyticus, Staphylococcus lugdunensis* and *Staphylococcus gallinarum* 1.47% each; and *Staphylococcus schleiferi, Staphylococcus warneri* and *Staphylococcus simulans* 0.74% each. The other genera identified were: *Escherichia coli* 10.29%; *Enterococcus faecalis* 6.61%; *Enterobacter aerogenes* 5.88%; *Streptococcus pyogenes* 2.94%; and *Citrobacter* sp.*, Salmonella* sp.*, Proteus mirabilis* and *Cedecea lapagei* 0.74% each. Among the microorganisms identified as agents causing vaginitis in the patients with lesions induced by HPV, *Staphylococcus aureus* was isolated as the most frequent agent in the women with lesions (6.66%) and the only agent in those without lesions (2.38%) (Table 1).

**Table 1:** Relative frequency of vaginal bacteria in patients with and without koilocytosis

Microorganism	Koilocytosis-positive	Koilocytosis-negative	Chi-squared	P
*Gardnerella vaginalis*	14 (23.3%)	4 (9.5%)	0.11	0.06
*Bacteroides* sp.	2 (3.3%)	1 (2.4%)	1.00	0.63
*Peptostreptococcus* sp.	1 (1.7%)	0 (0.0%)	1.00	0.58
*Prevotella sp.*	0 (0.0%)	1 (2.4%)	0.41	0.41
*Staphylococcus aureus*	21 (35.0%)	10 (23.8%)	1.46	0.22
*Enterococcus faecalis*	5 (8.3%)	4 (9.5%)	1.00	0.55
*Escherichia coli*	8 (13.3%)	6 (14.3%)	0.02	0.89
*Streptococcus agalactiae*	6 (10.0%)	1 (2.4%)	0.23	0.13

In the group with lesions, even though 50 (83.3%) of the patients said at the consultation that they suffered from vaginal discharges, only 17 of them (28.3%) satisfied all the diagnostic criteria for bacterial vaginosis. The vaginal pH was greater than 4.5 in 40 patients with lesions (66.6%), while the whiff test gave a positive result in 11 women (18.3%). Bacterial vaginosis diagnosed by means of cytology and Gram staining and culturing presented greater frequency in the group with koilocytosis than in the patients without lesions, although without statistical significance between the groups (Table 2).

**Table 2:** Prevalence of bacterial vaginosis and vulvovaginitis due to *Candida* sp. and *Trichomonas vaginalis* in patients with and without koilocytosis

Microorganism	Koilocytosis-positive	Koilocytosis-negative	Chi-squared	P	OR	CI (95%)	
Bacterial vaginosis	17 (28.3%)	6 (26.1%)	2.79	0.10	1.36	0.99	1.86
*Candida* sp.	27 (45.0%)	10 (23.8%)	4.79	0.02	1.43	1.05	1.95
*Trichomonas vaginalis*	6 (10.0%)	0 (0.0%)	4.46	0.04	1.78	1.49	2.12

*OR = odds ratio; CI = confidence interval.*

Among the microorganisms that are considered to cause bacterial vaginosis, we identified *Gardnerella vaginalis* as the most frequent agent in the patients with and without cell lesions. Positive diagnoses for vulvovaginitis via clinical and laboratory test methods were reported for 47.0% of the women investigated. This was more frequent among the koilocytosis-positive women (66.6%) and presented a statistically significant difference.

It could be seen that *Candida* sp. was the agent most frequently causing vulvovaginitis in the groups with and without koilocytosis: 25 patients (41.6%) and seven (17.5%), respectively. Thus, the patients with koilocytosis presented a statistically significant greater odds ratio for *Candida* sp. infection (Table 2). Through culturing, it was found that the species most frequently correlated with vulvovaginitis due to *Candida* sp. in the two groups were, in order: *C. albicans* 40.6%, *C. glabrata* 31.2%, *C. parapsilosis* 18.8% and *C. tropicalis* 9.4%. *Trichomonas vaginalis* was an agent that caused vulvovaginitis only in the women with koilocytosis, with a greater odds ratio for this group, as presented in Table 2.

From univariate analysis, the factors associated with the cell lesions were vaginal infections, use of oral contraceptives, alcoholism, number of sexual partners and nonuse of condoms. However, only the number of partners and condom use were independently associated with cell abnormalities in the multivariate analysis.

## DISCUSSION

The present study sought to determine the frequency of vulvovaginitis due to *Trichomonas vaginalis, Candida* sp. and bacterial vaginosis in women with and without koilocytosis, since there is an enormous empirical discussion regarding such an association. Various procedures were adopted in this study to minimize the occurrence of potential bias in collecting data from the patients. Moreover, concern for quality during sample collection guided the implementation of actions, with the aim of minimizing losses.

In the Pap test, it was found that smears presenting inflammation or abnormalities were more frequent among patients with koilocytosis than among those without it. Since koilocytosis usually represents the presence of human papillomavirus,^14-16^ it is believed that the presence of the virus would, in some manner, be a cofactor for infections by other pathogenic or opportunistic microorganisms.^17^ These opportunistic agents would be favored by imbalances in the vaginal flora.^18^

According to Edwards,^1^ *Escherichia coli* was the species most associated with bacterial vaginitis, although in the present series, it was found that *Staphylococcus aureus* was the agent most frequently causing it. Vaginal infections due to *Bacteroides* sp., *Peptostreptococcus* sp., *Prevotella* sp. and, particularly, *Gardnerella vaginalis* were also found to be present. The results were close to, but did not reach statistical significance, and this was probably because of the small sample size. Among the women without apparent genital infection, the obligatory anaerobic bacteria were isolated from 56% of them, with predominance of the genera *Bacteroides* sp. and *Peptostreptococcus* sp. These organisms are frequently involved in infections of the female genital tract, thus suggesting that the autochthonous vaginal microbiota is potentially pathogenic.^2^

Vulvovaginitis, whether sexually transmitted or not, was associated with koilocytosis in the present study. It was possible to demonstrate an association between *Candida* sp and *Trichomonas vaginalis* in the group of women who presented koilocytosis. A study on data from the 1960s through to the 1990s demonstrated that there has been a decrease in the frequency of cervicovaginal infection due to *Trichomonas vaginalis* and an increase in vulvovaginitis due to *Candida* sp., especially over the last decade.^19^

It is believed that bacterial vaginosis may in some manner be associated with the development of cervical intraepithelial neoplasia (CIN).^18^ It has been found that this is caused by risky sexual behavior and is secondary to sexually transmitted diseases or sexual promiscuity.^3^ In the present study, bacterial vaginosis was more frequently found in the group of women with koilocytosis, but without reaching statistical significance.

Infection by *Candida* sp. has been found in approximately 25% of patients with HPV,^11^ while our results showed a higher frequency of candidiasis among patients with koilocytosis. Although there is no confirmatory data in the literature, it is believed that one possible explanation for such an association is that infection by *Candida* sp. could activate the latent infection by HPV.^11,17^

The use of molecular biology may be capable of ascertaining associations between genetic and immunological factors and the presence of potentially pathogenic microorganisms.^20^

## CONCLUSIONS

On the basis of these results, it was found that the prevalence of fungal and parasitic vulvovaginitis seems to be higher in patients with koilocytosis, while the vaginal bacterial flora was not significantly associated with cell abnormalities.
